# DNA Analysis in Forensic Odontology: Applications, Challenges, and Comparative Effectiveness With Morphological Identification

**DOI:** 10.7759/cureus.109170

**Published:** 2026-05-19

**Authors:** Sreenitha S Hosthor, Sreelatha S, Shefali Sharma, Shahla Khan, Jay Gohil, Heena Tiwari

**Affiliations:** 1 Department of Forensic Odontology, Government Dental College and Research Institute, Bengaluru, IND; 2 Department of Oral Pathology, Specialist's Dental Clinic, Bengaluru, IND; 3 Department of Periodontics, Uttaranchal Dental and Medical Research Institute, Dehradun, IND; 4 Department of Oral Pathology, Dr. Ziauddin Ahmad Dental College and Hospital, Aligarh, IND; 5 Department of Prosthodontics, K.M. Shah Dental College and Hospital, Sumandeep Vidyapeeth (Deemed to be University), Vadodara, IND; 6 Department of Blood Cell, Commissionerate of Health and Family Welfare, Government of Telangana, Hyderabad, IND

**Keywords:** comparative effectiveness, dna, forensic, forensic odontology, morphological identification, narrative, odontology, review

## Abstract

Forensic odontology has traditionally relied on the morphological comparison of dental structures for human identification because of the durability and uniqueness of teeth. However, the emergence of DNA-based techniques has significantly enhanced the scope and reliability of forensic investigations, particularly in situations involving decomposed, burned, or fragmented remains, where conventional methods are limited. This scoping review aims to evaluate the applications of DNA analysis in forensic odontology, compare its effectiveness with that of morphological methods, and highlight current challenges and future directions. A structured review of literature published between 2000 and 2025 was conducted across major scientific databases, focusing on studies involving DNA extraction from dental tissues, profiling techniques, and comparative identification outcomes. Evidence indicates that dental pulp and dentin serve as reliable sources of DNA, with high success rates, even under adverse conditions. Short tandem repeat profiling remains the gold standard for individual identification, whereas mitochondrial DNA and next-generation sequencing expand applicability in degraded samples. Morphological methods continue to offer advantages in terms of speed, cost, and minimal infrastructure requirements, particularly when ante-mortem dental records are available. In contrast, DNA analysis demonstrates superior performance in challenging forensic scenarios such as mass disasters and advanced decomposition. Despite its advantages, DNA analysis has limitations, including degradation, contamination risk, high cost, and the need for specialized laboratory facilities. The ethical and legal considerations related to the use of genetic data remain significant. Overall, an integrated approach that combines morphological and molecular methods is the most effective strategy for human identification. Advances in rapid DNA technologies, sequencing methods, and standardization protocols are expected to further strengthen the role of forensic odontology in modern medical practice.

## Introduction and background

Forensic odontology plays a crucial role in human identification by utilizing dental structures that are highly resistant to environmental degradation and post-mortem changes. Traditionally, identification has relied on morphological comparisons between ante-mortem and post-mortem dental records, including radiographs, restorations, and anatomical features. These methods are widely accepted because of their cost-effectiveness, rapid application, and reliability in cases where adequate dental records are available [1,2]. However, their effectiveness is significantly reduced in situations involving severe thermal damage, advanced decomposition, or the absence of dental records, which are common in mass disasters and complex forensic scenarios [3].

DNA analysis has transformed forensic odontology by providing a highly sensitive and specific method for identification. Teeth act as protective reservoirs for genetic material, particularly in the pulp and dentine, enabling DNA recovery even under adverse conditions [4,5]. Techniques, such as short tandem repeat (STR) profiling, mitochondrial DNA analysis, and next-generation sequencing (NGS), have expanded the ability to obtain genetic profiles from compromised samples [6,7]. Despite these advances, challenges such as DNA degradation, contamination, infrastructure requirements, and ethical concerns persist, limiting its universal applicability.

Given the complementary strengths of morphological and molecular approaches, there is a growing need to systematically evaluate their comparative effectiveness and practical integration into forensic workflows. This review aims to evaluate the role of DNA analysis in forensic odontology and compare its effectiveness with that of conventional morphological identification methods. Specifically, this review sought to assess the various sources and methodologies of DNA extraction from dental tissues, examine the accuracy and applicability of different DNA profiling techniques, and critically compare DNA-based approaches with traditional morphological methods in terms of reliability, efficiency, and practical utility. Additionally, it aims to identify the key challenges, limitations, and emerging advancements in forensic dental identification, thereby highlighting future directions for improving the identification outcomes in diverse forensic scenarios.

## Review

Methodology

This review was conducted using a scoping review design to comprehensively map the available evidence on DNA analysis in forensic odontology and compare it with conventional morphological identification methods. A scoping approach was selected because of the diversity of study designs, methodologies, and outcome measures reported in the literature, making it suitable for summarizing a broad and evolving field, rather than performing quantitative synthesis. A structured literature search was performed across major electronic databases, including PubMed/MEDLINE, Scopus, and Web of Science. The search included studies published between January 2000 and March 2025 to capture advancements in modern DNA technologies relevant to forensic applications. Keywords and subject headings related to forensic odontology, dental DNA analysis, genetic profiling, and morphological identification have been used in various combinations. In addition, grey literature sources, such as disaster victim identification guidelines, forensic organization reports, and conference proceedings, were reviewed to ensure comprehensive coverage. Studies were selected based on predefined eligibility criteria as given in Table 1.

**Table 1:** Inclusion and exclusion criteria

Criterion	Inclusion	Exclusion
Study population	Human dental specimens; forensic casework; simulation studies	Animal models without human applicability discussion
Intervention/Exposure	DNA extraction, profiling, or sequencing from dental tissues	Studies exclusively on soft tissue or osseous DNA without dental focus
Comparator	Morphological dental comparison; other identification modalities	No comparator required for descriptive/methodological studies
Outcomes	Identification success rate, DNA yield, profile quality, comparative accuracy	Studies with no quantitative or qualitative outcome reporting
Study design	Experimental, observational, case series (n ≥ 5), reviews	Single case reports; opinion pieces without data
Language	English	Non-English without available translation

Data extraction was performed using a standardized approach to collect key information from each included study, including study characteristics, type of dental substrate, DNA analysis techniques used, and the main findings. The extracted data were synthesized narratively due to the heterogeneity of methodologies and outcomes, and no meta-analysis was performed.

Dental tissues as a source of DNA

Teeth represent one of the most reliable sources of DNA in forensic investigations because of their unique structural composition and resistance to environmental insults, such as decomposition, heat, and mechanical damage. The highly mineralized outer layers of the tooth protect the internal tissues, allowing the preservation of genetic material even under adverse conditions where other biological sources fail. Different dental components provide varying quantities and qualities of DNA, making an understanding of these substrates essential for effective forensic analysis [4,8].

Dental pulp is considered to be the most valuable source of nuclear DNA in teeth. It is a richly vascularized connective tissue containing numerous cellular elements, including fibroblasts and odontoblasts, which contribute to a high DNA yield. Studies have consistently demonstrated high success rates of DNA extraction from the pulp, particularly when the tooth structure remains intact. However, pulp tissue is susceptible to degradation due to thermal exposure, microbial activity, and environmental conditions, which may limit its availability in severely compromised remains [9].

Dentine serves as an important alternative source when pulp is degraded or absent. It contains microscopic tubules with remnants of cellular material and DNA bound to the mineral matrix, which provides protection against degradation. Although the DNA yield from dentin is generally lower than that from pulp, it has proven useful in cases involving long-term decomposition or historical samples. However, the extraction process requires demineralization, which is technically demanding [6,10].

Cementum contributes minimally to DNA yield due to its limited cellular content but may still provide usable genetic material under specific conditions. In contrast, enamel is highly mineralized and acellular, rendering it unsuitable for DNA extraction. A comparative overview of the DNA yield, success rates, and limitations across different dental substrates is presented in Table 2.

**Table 2:** Comparison of DNA yield across substrates [8-11]

Substrate	DNA yield (average)	Profile success rate	Optimal conditions	Limitations
Dental Pulp	High (500–2000 ng)	87–94%	Intact crown, <50 years post-mortem	Sensitive to heat, moisture, microbial activity
Dentine	Moderate (50–300 ng)	75–88%	Root intact, controlled environment	Requires demineralisation; lower copy number
Cementum	Low (<50 ng)	40–65%	Minimal exposure; dry environments	Very limited cell content
Bone (comparison)	Variable (100–800 ng)	60–72%	Dense cortical bone, dry conditions	More exposed to taphonomic insults
Enamel	Negligible	<5%	Not recommended	Virtually acellular

Overall, among dental tissues, pulp provides the highest yield and reliability, followed by dentin, while cementum offers limited utility and enamel is not considered a viable source for DNA analysis [11].

DNA extraction methodologies

**Figure 1:**
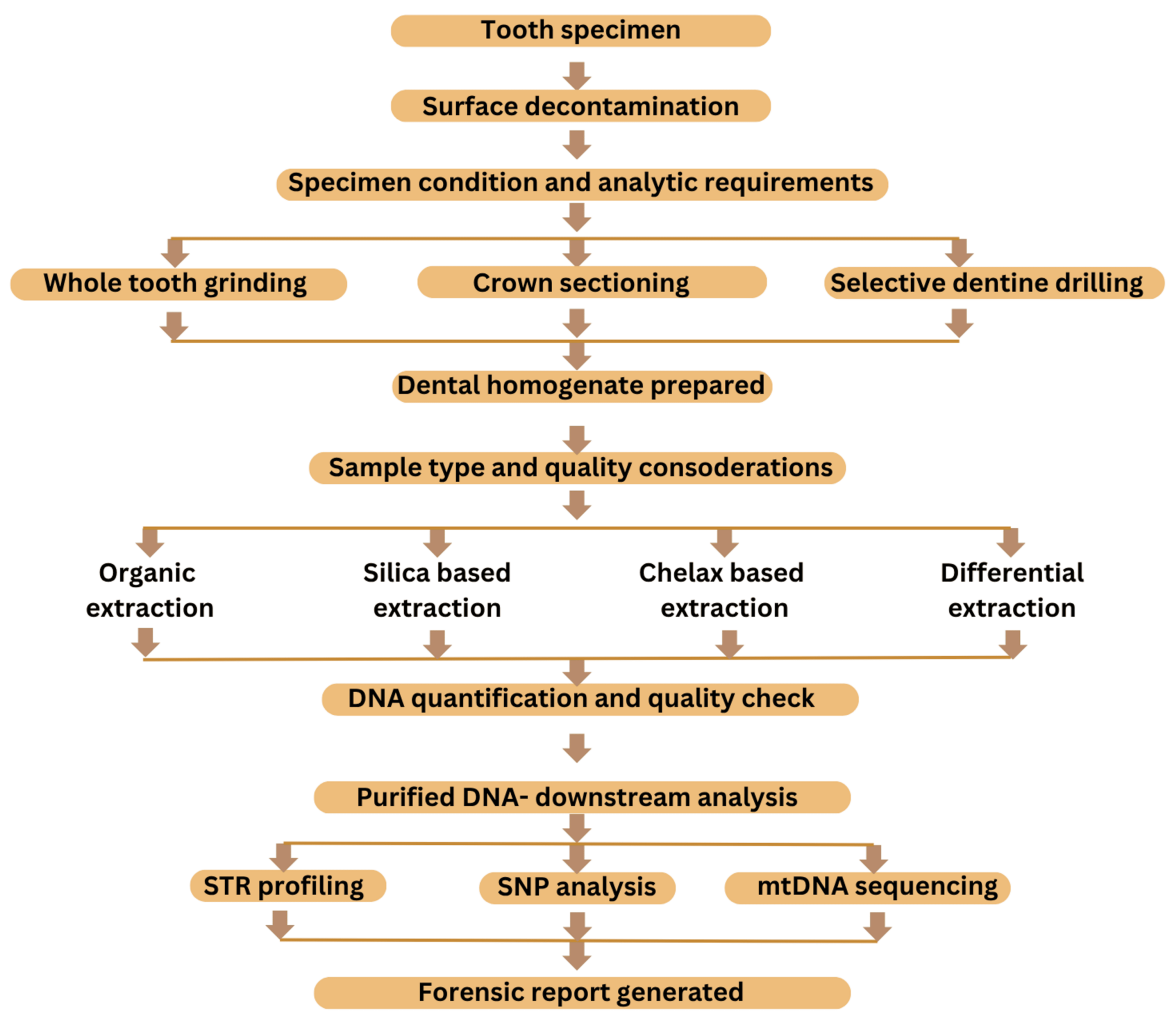
DNA extraction methodology STR: short tandem repeat; SNP: single nucleotide polymorphism; mtDNA: mitochondrial deoxyribonucleic acid. Image credit: Created by authors using Canva (Canva Pro, Canva Pty Ltd., Sydney, Australia).

DNA extraction from dental tissues is a critical step in forensic odontology, as it directly influences the quality and reliability of downstream genetic analyses (Figure 1).

The extraction process involves both physical preparation of the tooth and subsequent chemical or molecular procedures to isolate DNA, while minimizing contamination and inhibitors.

Sample preparation is the initial and crucial phase, with multiple approaches employed depending on the condition of the specimen and investigative requirements. Whole-tooth grinding involves cryogenic pulverization of the entire tooth after surface decontamination, allowing for maximum DNA recovery from all dental components [12]. However, this method results in the complete destruction of the specimen and is typically reserved for cases where morphological examination is not required. Alternatively, pulp isolation through crown sectioning enables direct access to the pulp chamber while preserving the tooth’s structural integrity. This approach is preferred when both morphological and molecular analyses are necessary [13]. In situations in which pulp tissue is absent or degraded, selective dentine drilling provides an effective alternative by targeting dentinal regions rich in residual DNA.

Following the sample preparation, various DNA extraction protocols were used. Organic extraction using phenol-chloroform has historically been considered the gold standard because of its high DNA yield and purity, although it is less commonly used today because of its complexity and use of hazardous chemicals [14]. Silica-based extraction methods have become the most widely adopted in forensic laboratories, offering efficient purification, compatibility with automation, and effective removal of inhibitors [15]. Chelex-based extraction provides a rapid and cost-effective alternative but is generally limited to samples with high DNA content [16]. In cases involving mixed biological samples, differential extraction techniques can be utilized to separate different cellular components prior to analysis.

A significant challenge in dental DNA extraction is the presence of polymerase chain reaction inhibitors such as humic substances, degraded proteins, and mineral components, which can interfere with amplification. Various strategies, including purification, dilution, and the use of inhibitor-resistant polymerases, have been employed to overcome these limitations [17]. Overall, the choice of extraction methodology depends on the condition of the sample, required DNA quality, and available laboratory resources, with silica-based methods currently representing the most practical and widely used approach for forensic applications.

DNA profiling techniques in forensic odontology

DNA profiling techniques play a central role in forensic odontology by enabling reliable human identification from dental tissues, particularly in cases in which conventional morphological methods are limited. Advances in molecular biology have introduced a range of techniques that vary in sensitivity, specificity, and applicability, depending on the quality and quantity of DNA obtained [11].

STR profiling is the gold standard for forensic identification. STRs are highly polymorphic regions of nuclear DNA that allow the generation of individual-specific genetic profiles with an extremely high discriminatory power. In forensic odontology, STR analysis of DNA extracted from dental pulp or dentin has demonstrated high success rates, even in degraded samples. Modified approaches, such as mini-STRs, which target shorter DNA fragments, have further improved the profiling success in compromised specimens [18,19].

Mitochondrial DNA (mtDNA) analysis is particularly valuable in cases in which nuclear DNA is insufficient or highly degraded. Owing to its high copy number per cell and maternal inheritance pattern, mtDNA can be recovered from challenging samples, including aged or environmentally exposed teeth [20]. However, its discriminatory power is lower than that of STR profiling because individuals sharing the same maternal lineage cannot be distinguished.

Y-chromosome STR (Y-STR) analysis provides additional utility in identifying male individuals and tracing paternal lineages. It is particularly useful in cases involving mixed DNA samples or when male-specific identification is required. Similar to mtDNA, its limitation lies in its reduced individual specificity among related male individuals [21].

NGS, also known as massive parallel sequencing, represents a significant advancement in forensic DNA analysis. NGS enables the simultaneous analysis of multiple genetic markers, including single nucleotide polymorphisms (SNPs), mtDNA, and STRs, offering enhanced sensitivity and the ability to analyze highly degraded DNA. It also supports emerging applications, such as forensic DNA phenotyping and ancestry inference, although its routine use is currently limited by cost and technical complexity [22,23].

Comparative effectiveness: DNA vs. morphological methods

Forensic identification relies primarily on two approaches: DNA-based analysis and traditional morphological dental comparisons. Each method has distinct advantages and limitations, and its effectiveness varies depending on the condition of the remains, the availability of reference data, and operational constraints. Morphological methods involve the comparison of postmortem dental findings with antemortem records, such as radiographs, restorations, and dental charts. These methods are rapid, cost-effective, and widely applicable when adequate records are available, and often provide reliable identification in routine forensic cases [4,5,8].

In contrast, DNA analysis offers superior discriminatory power and the ability to establish identity even in the absence of dental records. Techniques such as STR profiling provide highly specific individual identification, making DNA analysis particularly valuable in cases involving decomposed, burned, or fragmented remains [18,19]. However, DNA-based methods require specialized laboratory infrastructure, are relatively time-consuming, and incur higher costs than morphological approaches.

The effectiveness of these methods is highly scenario dependent. In cases of fresh remains with available dental records, morphological identification is often preferred owing to its efficiency and accessibility [24,25]. Conversely, in mass disasters, fire-related fatalities, or advanced decomposition, DNA analysis demonstrates clear advantages owing to its resilience to environmental degradation [26]. Importantly, both methods are complementary rather than competitive, and their combined application yields the highest identification success rate. A detailed comparison of the key parameters between the DNA and morphological methods is presented in Table 3.

**Table 3:** Performance comparison across key parameters STR: Short tandem repeat; mtDNA: Mitochondrial deoxyribonucleic acid; NGS: Next-generation sequencing; SWGDAM: Scientific Working Group on DNA Analysis Methods; OSAC: Organization of Scientific Area Committees; MFIs: Mixed forensic samples; Y-STR: Y-chromosomal short tandem repeat. [18,19,24-26]

Parameter	DNA analysis	Morphological methods
Identification power	Individual (STR: 1 in 10^18); population-level (mtDNA)	Individual: dependent on dental uniqueness and record quality
Speed	4–96 hours (lab-based); 90 min (rapid DNA)	Hours to days
Cost per case	USD 300–2,500 (STR); higher for NGS	USD 50–400
Requirement for reference	Biological reference from individual or relative	Antemortem dental records
Performance with degraded remains	High (particularly dentine; mtDNA for severely degraded)	Low to moderate
Performance with fire/explosion victims	Moderate (pulp protection by crown)	Low (morphological distortion)
Performance in MFIs	High: scalable with automation	High: scalable with trained teams
Exclusion capability	Definitive genetic exclusion	Exclusion by non-correspondence of unique features
Infrastructure required	Molecular laboratory, validated equipment	Dental operatory, light source, magnification
Legal admissibility	Widely accepted; SWGDAM/OSAC standards	Accepted; subjective elements debated
Age/sex estimation	Limited (Y-STR for sex; methylation for age)	Established methods (wear, root transparency, etc.)

Challenges and limitations

Despite significant advancements in DNA analysis within forensic odontology, several technical, operational, and ethical challenges continue to limit its universal applicability. One of the primary concerns is DNA degradation, which occurs because of environmental factors such as heat, moisture, microbial activity, and prolonged postmortem intervals [27,28]. Degraded DNA often results in fragmented genetic material, leading to incomplete profiles, allelic dropout, or amplification failure, particularly in conventional STR-based techniques.

Another major limitation is the presence of polymerase chain reaction (PCR) inhibitors in the dental samples. Substances such as humic acids, degraded proteins, and mineral components of teeth can interfere with the amplification processes, reducing the reliability of DNA profiling. Although modern extraction and purification techniques have improved inhibitor removal, this remains a significant technical challenge [29].

Contamination is an additional critical concern, particularly in forensic settings that involve multiple handlers or mass disaster scenarios. Contamination can occur during sample collection, processing, or analysis, potentially leading to erroneous results. Strict adherence to contamination control protocols, including the use of dedicated laboratory spaces and elimination databases, is essential to mitigate this risk [30].

From an operational perspective, DNA analysis requires specialized laboratory infrastructure, trained personnel, and relatively high financial investment, which may not be readily available in all settings, especially in resource-limited regions. This creates disparities in the applications of advanced forensic techniques across different geographical areas.

Furthermore, limitations in population databases can affect the statistical interpretation of DNA profiles, particularly in underrepresented populations. Ethical and legal concerns also arise regarding the storage, use, and privacy of genetic information, especially in cases involving familial searching or forensic DNA phenotyping [31,32]. Overall, although DNA analysis has significantly enhanced the capabilities of forensic odontology, these challenges highlight the need for standardized protocols, improved infrastructure, and careful ethical governance to ensure accurate and responsible application in forensic practice.

Rapid DNA technology has emerged as a promising tool for forensic applications, enabling automated DNA profiling in a short period of time without extensive laboratory infrastructure. While currently optimized for soft tissues, ongoing developments aim to adapt these systems to dental samples, particularly in mass disaster scenarios. Overall, the selection of DNA profiling techniques depends on the sample conditions, availability of reference material, and the required level of discrimination. A combined and tiered approach using multiple techniques can enhance the accuracy and reliability of forensic dental identification.

Emerging technologies and future directions

Recent advancements in molecular biology and digital technologies have significantly expanded the scope of forensic odontology, thereby enhancing both the accuracy and applicability of DNA-based identification. Emerging techniques aim to overcome the limitations of conventional methods, particularly in cases involving degraded samples or absence of reference data.

Forensic DNA phenotyping (FDP) is an evolving approach that enables the prediction of externally visible characteristics such as eye color, hair color, skin pigmentation, and ancestry from genetic material [33]. This technique provides valuable investigative leads in cases in which direct identification is not possible, although its predictive accuracy and ethical implications require careful consideration. Epigenetic analysis, particularly DNA methylation profiling, has shown promise for estimating the biological age of dental tissues. Age-related changes in methylation patterns can provide relatively accurate age estimates, independent of morphological features, making this approach especially useful in cases where dental structures are compromised [34].

NGS technologies continue to advance forensic DNA analysis by enabling high-throughput sequencing of multiple genetic markers, including single nucleotide polymorphisms and mitochondrial DNA [23]. These techniques improve the sensitivity of degraded samples and allow for more comprehensive genetic profiling, including kinship analysis and ancestry inference. Microbiome analysis represents another emerging area where the oral microbiota associated with dental tissues may provide supplementary information regarding an individual’s lifestyle, geographic origin, or health status [35]. Although still in its early stages, this approach may complement traditional identification methods in complex cases.

Artificial intelligence (AI) and machine learning are increasingly being integrated into forensic odontology for automated analysis of dental radiographs and morphological features. These tools have the potential to improve efficiency, reduce observer bias, and enhance the reproducibility of the identification processes [36]. Additionally, the development of portable and rapid DNA technologies offers the possibility of on-site genetic analysis in mass disaster scenarios, significantly reducing turnaround time and improving operational efficiency [25,26]. Overall, these emerging technologies highlight a shift toward a more integrated, multidisciplinary approach in forensic odontology. Continued research, validation, and ethical oversight are essential to ensure reliable and responsible application in forensic practice.

An integrative identification framework

A growing body of evidence supports the need for an integrated, tiered approach to human identification in forensic odontology, rather than relying on a single modality. Both DNA-based and morphological methods possess distinct advantages, and their combined application enhances overall identification accuracy and efficiency across diverse forensic scenarios.

An integrative framework allows for the systematic selection of identification methods based on case-specific factors such as the condition of remains, availability of antemortem records, and resource accessibility. Morphological comparison remains the first-line approach in cases involving well-preserved dental structures and available dental records owing to its rapidity and cost-effectiveness. However, when morphological methods are inconclusive or infeasible, DNA analysis is a highly reliable secondary approach, providing definitive identification through genetic profiling.

In more challenging scenarios, such as advanced decomposition, thermal damage, or the absence of direct reference samples, alternative DNA techniques, including mtDNA analysis, mini-STRs, and NGS, can be employed [20,22,27]. These methods extend the applicability of forensic identification to degraded or complex cases, albeit with limitations in discriminatory power or resource requirements. This tiered strategy ensures the optimal utilization of available techniques while minimizing unnecessary expenditure of time and resources. Furthermore, the integration of emerging technologies such as rapid DNA systems and AI is expected to streamline forensic workflows. A structured overview of this tiered identification approach is presented in Table 4.

**Table 4:** Tiered approach. STR: Short tandem repeat; DNA: Deoxyribonucleic acid; mtDNA: mitochondrial deoxyribonucleic acid; NGS: Next-generation sequencing; LCN: Low copy number; aDNA: Ancient deoxyribonucleic acid. [20,22,27]

Tier	Approach	Indication	Expected outcome
Tier 1	Morphological comparison	Fresh/recent remains; dental records available; identifiable dental anatomy	Rapid identification in 60–70% of cases in record-rich environments
Tier 2	STR profiling (nuclear DNA)	Morphological comparison inconclusive/impossible; biological reference available	Individual identification; resolves equivocal morphological findings
Tier 3	Mini-STR / mtDNA analysis	Degraded specimens; STR profiling fails; maternal reference only	Partial profiles; population-level inclusion/exclusion
Tier 4	NGS / LCN / aDNA techniques	Severely degraded; ancient remains; all preceding tiers fail	Probabilistic identification; ancestry; phenotypic prediction

## Conclusions

DNA analysis has significantly advanced forensic odontology by enabling reliable human identification in challenging conditions, such as decomposition, thermal damage, and mass disasters. Dental tissues, particularly pulp and dentin, serve as protected reservoirs of DNA, allowing successful profiling, even in compromised samples. Although STR profiling remains the gold standard, emerging technologies continue to enhance their diagnostic capabilities. However, DNA analysis complements rather than replaces morphological methods, which remain rapid, cost-effective, and highly effective when ante-mortem records are available. Challenges such as DNA degradation, contamination, and resource limitations persist and must be addressed through standardization and improved infrastructure. Overall, a combined, tiered approach that integrates both morphological and molecular techniques is the most effective and practical strategy for forensic identification.
